# Mobile Phone Text Messages to Support People to Stop Smoking by Switching to Vaping: Codevelopment, Coproduction, and Initial Testing Study

**DOI:** 10.2196/49668

**Published:** 2023-09-27

**Authors:** Vassilis Sideropoulos, Eleni Vangeli, Felix Naughton, Sharon Cox, Daniel Frings, Caitlin Notley, Jamie Brown, Catherine Kimber, Lynne Dawkins

**Affiliations:** 1 Department of Psychology & Human Development IOE, UCL’s Faculty of Education and Society University College London London United Kingdom; 2 Division of Psychology London South Bank University London United Kingdom; 3 School of Health Sciences University of East Anglia Norwich United Kingdom; 4 Department of Behavioural Science and Health University College London London United Kingdom; 5 Norwich Medical School University of East Anglia Norwich United Kingdom

**Keywords:** coproduction, SMS text messages, e-cigarette, smoking, eHealth, vaping, mobile phone, codevelopment, text message

## Abstract

**Background:**

SMS text messages are affordable, scalable, and effective smoking cessation interventions. However, there is little research on SMS text message interventions specifically designed to support people who smoke to quit by switching to vaping.

**Objective:**

Over 3 phases, with vapers and smokers, we codeveloped and coproduced a mobile phone SMS text message program. The coproduction paradigm allowed us to collaborate with researchers and the community to develop a more relevant, acceptable, and equitable SMS text message program.

**Methods:**

In phase 1, we engaged people who vape via Twitter and received 167 responses to our request to write SMS text messages for people who wish to quit smoking by switching to vaping. We screened, adjusted, refined, and themed the messages, resulting in a set of 95 that were mapped against the Capability, Opportunity, and Motivation–Behavior constructs. In phase 2, we evaluated the 95 messages from phase 1 via a web survey where participants (66/202, 32.7% woman) rated up to 20 messages on 7-point Likert scales on 9 constructs: being understandable, clear, believable, helpful, interesting, inoffensive, positive, and enthusiastic and how happy they would be to receive the messages. In phase 3, we implemented the final set of SMS text messages as part of a larger randomized optimization trial, in which 603 participants (mean age 38.33, SD 12.88 years; n=369, 61.2% woman) received SMS text message support and then rated their usefulness and frequency and provided free-text comments at the 12-week follow-up.

**Results:**

For phase 2, means and SDs were calculated for each message across the 9 constructs. Those with means below the neutral anchor of 4 or with unfavorable comments were discussed with vapers and further refined or removed. This resulted in a final set of 78 that were mapped against early, mid-, or late stages of quitting to create an order for the messages. For phase 3, a total of 38.5% (232/603) of the participants provided ratings at the 12-week follow-up. In total, 69.8% (162/232) reported that the SMS text messages had been useful, and a significant association between quit rates and usefulness ratings was found (*χ*^2^_1_=9.6; *P*=.002). A content analysis of free-text comments revealed that the 2 most common positive themes were *helpful* (13/47, 28%) and *encouraging* (6/47, 13%) and the 2 most common negative themes were *too frequent* (9/47, 19%) and *annoying* (4/47, 9%).

**Conclusions:**

In this paper, we describe the initial coproduction and codevelopment of a set of SMS text messages to help smokers stop smoking by transitioning to vaping. We encourage researchers to use, further develop, and evaluate the set of SMS text messages and adapt it to target populations and relevant contexts.

## Introduction

### Background

e-Cigarettes, also known as vapes, have become a popular choice among individuals attempting to quit smoking, particularly in England [1]. There is a growing body of evidence supporting the efficacy of vapes in helping individuals quit smoking [2,3], as well as demonstrating the reduced exposure to toxins and improved health outcomes for those who fully switch to vaping [4,5]. However, despite their popularity, it has been observed that approximately half of the people who vape continue to smoke tobacco (dual use). Many others have attempted to use vapes but have not continued to use them [6]. The lack of satisfaction is cited as a major reason for tobacco relapse or discontinuation of vape use [6]. Perceptions of harm and safety concerns [6,7], concern about continued addiction [6], practical or technical difficulties [6], difficulties finding the right device [8,9], and inadequate craving relief [6,8] are other commonly cited reasons. This suggests that more could be done to improve confidence in the products and support people who smoke in using vapes optimally to quit smoking. Although nicotine is not devoid of risk, smoking is a primary contributor to premature mortality. Thus, shifting individuals away from smoking is a core objective. Aligning with the UK National Institute for Health and Care Excellence guidelines [9], our SMS text messages highlight the use of nicotine vapes for relapse prevention. The National Institute for Health and Care Excellence guide advocates for the extended use of licensed nicotine-containing products to avert relapse in line with our SMS text message program’s approach.

Although there is interpersonal advice and support for those interested in switching from smoking to vaping (ie, in vape shops and Stop Smoking Services [10]), there is a paucity of concurrent support for those who purchase vapes via the web. Consumers may be transitioning from smoking to vaping without knowing the technicalities of the device, flavor, and nicotine strengths. Mobile phone SMS text messaging may be one way to improve outcomes and is cheap and easy to implement, but coproduction with people who vape is essential to ensure the relevance of the messages. The impact of coproduction paradigms in research is vast—they (1) enable the targeting of embedded community needs and engaging of community stakeholders in meaningful and authentic rigorous research that incorporates diverse perspectives, experiences, and knowledge; (2) promote an equitable practice (eg, people who are affected by services and outcomes of research); and (3) have a patient-centered focus in the design of interventions [11,12]. Overall, the ethos of coproduction fosters an inclusive research paradigm by promoting equality in shared decision-making and mutual respect between researchers and community stakeholders, which builds trust and addresses the power imbalances that exist in traditional research approaches.

In the general smoking cessation field, SMS text messages have been found to be an effective tool for smoking cessation and can increase abstinence rates [13,14]. In a recent systematic review, SMS text message interventions were more effective than minimal smoking cessation support across 13 studies (risk ratio 1.4), and there was also evidence that adding SMS text messaging to other smoking cessation interventions improved abstinence rates compared with smoking cessation interventions alone (risk ratio=1.6) [13]. However, there are currently no SMS text message interventions that have been specifically designed to assist people who smoke to stop smoking by switching to vaping.

Intervention design should draw on behavioral theory to identify the mechanisms of action that lead to behavior change [15,16]. This project used the Behavior Change Wheel [17] to provide a comprehensive framework for this purpose, offering tools that can be used to specify both the characterization of intervention content and the theoretical mechanisms of action. At the core of the Behavior Change Wheel is the Capability, Opportunity, and Motivation–Behavior (COM-B) model [17], which postulates that 3 essential conditions are necessary for behavior change. According to the theory, behaviors only occur when the individual has the psychological and physiological “capability” as well as the social and physical opportunity to engage in them and has more reflective or automatic motivation to enact them than other competing behaviors at any given moment. To be successful, behavior change requires sustained change in one or more of these conditions. Therefore, the intervention content is designed to address these conditions.

The Behavior Change Technique Taxonomy version 1 [18] identifies 93 behavior change techniques (BCTs) to enable the characterization of the behavior change content of interventions. BCTs are the smallest components of an intervention that have the potential to change behavior (ie, the “active ingredients”) by targeting the different elements of the COM-B model, aiding in the selection and implementation of effective behavior change strategies.

### Objectives

In this paper, we describe how we worked with people who vape and smoke (or who previously vaped and smoked) to develop, evaluate, and test a set of SMS text messages that could be used by other researchers across a range of smoking cessation studies using vapes. This process was divided into 3 distinct phases.

In phase 1, the focus was on generating SMS text messages through the input and perspectives of current and former people who vape and smoke. This phase aimed to capture the unique experiences of these individuals to create SMS text messages that were tailored to their specific needs and challenges. Finally, theoretical frameworks and clinical expertise of the research team were applied to the SMS text messages.

In phase 2, the recommendations from phase 1 were evaluated against several concepts for suitability to users. This phase aimed to ensure that the SMS text messages were evidence-based and aligned with the current practices in smoking cessation.

Finally, in phase 3, the SMS text messaging program was used as part of a larger web-based randomized optimization study [19]. The larger study aimed to identify the most effective ways (including SMS text messaging) of supporting smokers to quit smoking by switching to an e-cigarette.

## Methods

### Overview

The selection of the SMS text messages for the program was informed by a coproduced examination of the COM-B constructs and BCTs used in smoking cessation interventions followed by an application of the final set of SMS text messages in a randomized optimization trial [19,20]. Phases 1 and 2 took place before the COVID-19 pandemic. However, phase 3 coincided with the COVID-19 pandemic.

### Ethical Considerations

Phase 2 and phase 3 were approved by the School of Applied Sciences Research Ethics Committee at London South Bank University (ETH1819-0143 and ETH1920-0043, respectively). Informed consent was obtained from all participants. Privacy and confidentiality were achieved by anonymizing all the data. Participants received no compensation for taking part in the project at phase 2, and in phase 3, participants were reimbursed with a £10 (US $12.68) Amazon voucher.

### Phase 1: Text Generation and Development

#### Text Generation

Between January 2019 and March 2019, the following tweet was posted on Twitter: “Vapers please help! We are developing new text messages to help ‘would be’ vapers make the switch. What advice would you give in a text message?” A total of 151 SMS text message recommendations were received. An additional 16 suggestions were made in a thread posted on “Planet of the Vapes” (a popular vaping web-based information forum).

#### Text Development

All authors reviewed the suggestions received. In total, 35.9% (60/167) of the suggestions were removed, mainly as they were descriptive accounts of participants’ own experiences rather than advice per se. Other duplicate messages and those with inappropriate content were also removed. The remaining messages (n=42) were refined or reworded, and the character count was reduced where necessary to ensure that the messages were short and adhered to the 160-character limit for a single SMS text message. A total of 53 messages were also added by the authors at this stage based on their own research or clinical work and professional experience, resulting in a set of 95 messages. The authors then grouped the messages into key themes through an iterative process of discussion, categorization, and consensus. In total, 7 themes were identified: smoking cessation support, social and practical support, identity, preventing lapses and relapses, vaping versus smoking, practical vaping tips (equipment), and health and safety.

To support the use of theory and mechanisms of action in intervention design, the 95 SMS text messages were mapped against the COM-B behavior change constructs and BCTs. Some of the texts contained links to videos, but as the intervention content was external to the message and engagement was optional, these were not coded. In total, 2 authors (LD and EV) identified the behavior or behaviors and condition or conditions targeted in each SMS text message, and these were then independently examined by FN, CN, and VS. Coders met to discuss discrepancies and refine the mapping strategy, for example, coding behavioral substitution only where the text explicitly referred to replacing smoking (the unwanted behavior) with vaping (the desired behavior), as well as the blanket application of BCT pharmacological support to all texts where vape use was the behavior (as vaping is pharmacological support in itself). The revised coding was applied following a similar process to the original coding, and any remaining discrepancies were further discussed and resolved.

### Phase 2: Assessment and Refinement of Text Messages

#### Assessment of Text Messages

The 95 SMS text messages from phase 1, along with 49 generic smoking cessation SMS text messages from the iQuit in Practice study [21] (for a separate project; data not included in this paper), were presented in a Qualtrics (Qualtrics International Inc) survey to people who smoke or vape. Each participant rated up to 20 SMS text messages randomly generated from each of the 7 themes on a 7-point Likert scale for the following constructs: how clear, understandable, believable, and helpful for smokers they considered the SMS text messages to be and whether they would be happy to receive them (with a higher score indicating a more favorable response). They also rated how the text made them feel: positive (1) versus negative (7), offended (1) versus unoffended (7), enthusiastic (1) versus unenthusiastic (7), and interested (1) versus uninterested (7). In addition to these 9 constructs, a free-text box for optional comments was available. We received 61 comments with recommendations, feedback, and suggestions for new SMS text messages or rewording. The full data set and syntax are available in a web-based repository [22].

#### Data Analysis Plan

Descriptive statistics were used to generate a total score across the 9 different constructs, followed by a computation of the mean across participants. Any SMS text message that received a mean score of ≥4 was included in the final set of SMS text messages.

#### Refinement of Text Messages

A working group (LD, SC, and 2 people with extensive vaping experience) reviewed and discussed the survey results, including messages with low ratings and comments or suggestions from survey participants. Further amendments were made to wording following advice about the use of consistent and understandable wording or terminology.

Finally, a second working group (LD and SC with 2 further individuals with vaping experience) matched the messages to the early, mid-, or late stages of quitting or vaping and created a suggested schedule, ensuring adequate temporal representation of each theme and COM-B construct. A total of 2 messages were added at this stage to introduce and conclude the set of messages, producing a final set of 78 messages, which are available for other researchers to use in smoking cessation studies.

### Phase 3: Application of the Final Text Messages Set in a Randomized Optimization Trial

The third phase required the application and evaluation of the selected intervention SMS text messages through a planned randomized optimization trial.

#### Overview of Participants and Procedure of the Randomized Optimization Trial

A total of 603 participants (mean age 38.33, SD 12.88 years; n=369, 61.2% women; n=239, 39.6% men; and n=1, 0.2% nonbinary individuals) were randomized to receive SMS text message support as part of a web-based randomized optimization study described elsewhere [20]. All participants received a voucher to purchase an e-cigarette starter kit via a website, and they were sent a link to complete a follow-up survey after 12 weeks. Those who were allocated to the SMS text message condition were sent 72 messages twice daily for the first 2 weeks; once a day for the following 4 weeks; then every other day for 4 weeks; and, finally, once a week for 2 weeks. The full data set and syntax are available in a web-based repository [23].

#### Data Analysis Plan

For phase 3, the aim was to evaluate the application of the final SMS text messages in the randomized optimization trial. A set of questions was asked at the 12-week follow-up regarding the usefulness, frequency, and perception of the SMS text messages. In terms of the quantitative data, we were interested in the responses from people who answered the questions about their experiences of receiving texting support. To do so, we computed the percentages for usefulness, frequency, and whether they had blocked the messages. This was followed by an association test (a chi-square test), which explored perceptions of the usefulness of the SMS text messages and self-reported abstinence status (not a single puff in the last 4 weeks at the 12-week follow-up point).

Participants also had the opportunity to provide feedback on the SMS text message program. To understand the feedback provided, a content analysis approach was used.

## Results

The codevelopment resulted in an SMS text message program that was focused on supporting people in quitting smoking using an e-cigarette over a 12-week period.

### Phase 1: Examination of the Generated Text Messages and Development of an Initial Set

Multimedia Appendix 1 shows the 95 SMS text messages mapped according to COM-B constructs. In total, 3 behaviors are targeted by the SMS text messages. Practical advice on how to use the vape was the most common behavioral target, followed by smoking cessation and purchasing vape equipment.

### Phase 2: Assessment and Refinement of the Initial Set of Text Messages

#### Participants

A total of 202 participants (mean age 48.31, SD 12.63 years; 66/120, 55% woman) completed a web-based survey. Most participants (95/121, 78.5%) vaped daily and were ex-smokers (90/121, 74.4%). Table 1 presents a detailed demographic overview of our sample.

The participants were recruited through social networks (Twitter and Planet of the Vapes) and word of mouth. Anyone aged >18 years who vaped, smoked, or used both and who was fluent in English was eligible for the study.

**Table 1:** A detailed overview of the participants’ demographics (N=202).

Demographics			Values
Age (years), mean (SD)			48.31 (12.63)
**Gender, n (%)**			
	Woman	66 (55)	
	Man	54 (45)	
	Missing	82 (40.6)	
**Ethnicity, n (%)**			
	Asian and Asian British	1 (0.8)	
	White	115 (95)	
	Mixed race	1 (0.8)	
	Multiple ethnic groups	2 (1.7)	
	Other	2 (1.7)	
	Missing	81 (40.1)	
**Smoking status, n (%)**			
	Yes, daily	21 (17.4)	
	Yes, nondaily	10 (8.3)	
	No	90 (74.4)	
	Missing	81 (40.1)	
**Vaping status, n (%)**			
	Yes, daily	95 (78.5)	
	Yes, nondaily	10 (8.3)	
	No	16 (13.2)	
	Missing	81 (40.1)	

#### Assessment Outcomes of the Initial Set of Text Messages

Mean scores were computed for each message across all 9 construct ratings. Multimedia Appendix 1 presents all 9 construct ratings combined with the message primary theme and COM-B construct. For this analysis, we only included participants who provided 1 complete set of construct ratings for at least one text.

The highest-rated SMS text message for each theme against each rating is shown in Multimedia Appendix 1. In relation to the individual dimensions rated, all SMS text messages received a mean score higher than the neutral anchor of 4 (our specified threshold for inclusion) on how clear, understandable, and believable they were perceived to be. A total of 9 SMS text messages received construct ratings outside of the prespecified threshold for inclusion on some of the other constructs: 2 (22%) of these had unfavorable scores (ie, above or below the neutral anchor of 4) across multiple constructs, so they were removed (Multimedia Appendix 1). The other 7 were discussed with 2 people who vape, and 6 (86%) of these were retained. On the basis of the feedback we received from the survey, 24 SMS text messages were also removed (eg, because of a lack of appeal to specific groups such as older people, confusion, or ostensible repetition of the same theme), and 7 new messages (based on suggestions from participants or vapers in our working group) were added, resulting in 76 messages.

Multimedia Appendix 2 shows the final set of messages in the suggested order with the target behavior, COM-B construct, and BCTs. Across the final set of messages, a total of 15 BCTs and 96 uses of these BCTs were identified. Most BCT uses were from the shaping knowledge (26 messages), regulation (22 messages), natural consequences (19 messages), self-belief (10 messages), and social support (8 messages) groups. There was coverage across all COM-B constructs in the program. The most frequent COM-B constructs, representing potential mechanisms of action, were reflective motivation (30 messages), psychological capability (23 messages), physical capability (12 messages), physical opportunity (7 messages), social opportunity (5 messages), and automatic motivation (4 messages).

### Phase 3: Outcomes of the Application of the Final Set of Text Messages

For phase 3, our participants were recruited through social networks (Twitter and Reddit) and word of mouth. Anyone aged >18 years who was interested in switching to vaping to quit smoking and was fluent in English was eligible for the study. The randomized controlled trial study contains more information [19].

A total of 38.5% (232/603) of the participants responded to at least one of the SMS text message questions at the 12-week follow-up. In total, 69.8% (162/232) reported that they found the messages useful, 7.8% (18/232) reported that they were not useful, and 6.9% (16/232) stated that they had not received the texts. A total of 66.2% (143/216) stated that the message frequency was about right, 32.4% (70/216) stated that the messages were too frequent, and 1.4% (3/216) reported that the messages were not frequent enough. In total, 6.5% (14/216) reported blocking the messages as they were too frequent, too annoying, or too repetitive (based on 8 comments).

A total of 20.3% (47/232) of the participants provided further comments, which we analyzed using content analysis. A coding frame was first agreed upon for the classification of comments as positive, negative, mixed (containing both positive and negative points), or ambiguous (used when a comment was unclear, eg, because of a typographical error). LD and CN then independently applied the coding framework deductively. A total of 45% (21/47) of the comments were coded as positive, 34% (16/47) were coded as negative, 15% (7/47) were coded as mixed, and 6% (3/47) were coded as ambiguous. Next, LD and CN independently inductively coded the content of the comments to identify categories. The most common positive categories were *helpful* (13/47, 28%), *encouraging* (6/47, 13%), *informative* (4/47, 9%), and *motivating* (4/47, 9%), and the most common negative categories were *too frequent* (9/47, 19%), *annoying* (4/47, 9%), *could not block* (4/47, 9%), and *useless* (3/47, 6%). Finally, those who rated the SMS text messages as useful were more likely to quit than those who rated them as not useful (*χ*^2^_1_=9.6; *P*=.002).

The list of codes, frequencies, and Cohen κ values is shown in Table 2. There was 100% agreement between the 2 coders for the classification of comments as positive, negative, mixed, or ambiguous (weighted κ=1 in all cases). In relation to the categories, weighted κ coefficients for all categories in the initial coding were between substantial and perfect (range 0.66-1) apart from 3 disagreements (in which κ values could not be computed because of a single instance of the categories by 1 coder). These inconsistencies were reviewed and discussed, and the coding frame was revised to reduce the categories and allow for more interpretation of the concepts.

**Table 2:** Ratings of constructs against the SMS text messages (n=47).

Classification			Participants, n (%)		Cohen weighted κ (95% CI)
Positive			21 (45)		1 (1-1)
Negative			16 (34)		1 (1-1)
Mixed			7 (15)		1 (1-1)
Ambiguous			3 (6)		1 (1-1)
**Positive categories**					
	Helpful	13 (28)		0.88 (0.74-1.04)	
	Encouraging	6 (13)		1 (1-1)	
	Informative	4 (9)		1 (1-1)	
	Motivating	4 (9)		0.87 (0.64-1.11)	
	Supportive	3 (6)		1 (1-1)	
	Great, excellent, or good	3 (6)		0.79 (0.39-1.19)	
	Enjoyable	3 (6)		1 (1-1)	
	Missed when stopped	1 (2)		1 (1-1)	
	Stayed on track	1 (2)		1 (1-1)	
	Relevant	1 (2)		1 (1-1)	
	Factual	1 (2)		1 (1-1)	
	Well timed	1 (2)		1 (1-1)	
	Positive impact	1 (2)		1 (1-1)	
	Unbiased	1 (2)		1 (1-1)	
**Negative categories**					
	Too frequent	9 (19)		0.76 (0.51-1.02)	
	Annoying	4 (9)		1 (1-1)	
	Could not block	4 (9)		0.73 (0.37-1.09)	
	Useless	3 (6)		1 (1-1)	
	Patronizing	2 (4)		1 (1-1)	
	Ignored	2 (4)		0.66 (0.03-1.23)	
	Repetitive	2 (4)		1 (1-1)	
	Triggering	2 (4)		1 (1-1)	
	Badly timed	2 (4)		1 (1-1)	
	Bored	1 (2)		1 (1-1)	
	Deleted	1 (2)		1 (1-1)	
	Wrong context	1 (2)		1 (1-1)	
	Not required	1 (2)		1 (1-1)	
	Blocked	1 (2)		1 (1-1)	

## Discussion

### Principal Findings

Coproduction in health research is increasingly becoming a valued approach as it has the potential to improve the relevance, quality, and impact of research outcomes [24]. Through this approach, the expertise and knowledge of researchers, health care providers, and the community are recognized [12,24]. In this study, we used a coproduction paradigm to codevelop and coproduce a mobile phone SMS text message program with ex-smokers and people who vape that aimed to support people in quitting smoking by switching to vaping. The significance of this work lies in the representation of the voices of those who quit smoking by using a vape. In addition, our work ensures that our SMS text message program is driven by a community that has direct experiences of attempts to quit and does not solely rely on scientific evidence that often does not encapsulate the diverse voices of people.

In the context of codeveloping a mobile phone SMS text message program, we worked with community stakeholders in phase 1 to identify SMS text messages that could support people to quit smoking by switching to vaping. We received 151 SMS text message recommendations that captured different perspectives and experiences of the community members. By combining the diverse perspectives of the community and our expertise in the field, we attempted to capture a more relevant, feasible, and acceptable focus on topics that people who wish to quit smoking by switching to a vape would consider. Despite the high volume of recommendations and the different views from the community, the working group of researchers and people who vape invested substantial amounts of time in sorting and reviewing the SMS text messages for several reasons (eg, difference in opinions, inaccurate medical information, and shortening of the messages to fit in a single 160-character SMS text message). This highlights the substantial time and resources required to enable a full coproduction paradigm, which can also be a barrier to its implementation [11]. Similarly, in phase 2, further review was required by our working group for the refinement of the final mobile phone SMS text message program. Although the input from the community is invaluable, the limits of coproduction must be considered.

SMS text message interventions for smoking cessation have been found to be efficient, convenient, and cost-effective [14] as well as improving smoking abstinence rates [25] because of the personalized support and reminders the smokers receive [26,27]. Although our SMS text messages were not personalized to individuals, they were coproduced with people who had experienced the transition from smoking to vaping and were tailored to the stage in that journey; thus, our study meets a gap in the provision of SMS text messages to specifically support smoking cessation using an e-cigarette device. In addition to providing a program of SMS text messages to use in intervention design, specification according to BCTs, with linking to mechanisms of action, offers opportunities for the development of interventions that can test hypotheses about the theoretical underpinning of these via the conduct of mediational analyses.

The strength of our study lies in the coproduction and codevelopment of the SMS text messages. Through the implementation of the mobile phone SMS text message program in phase 3, we had the opportunity to obtain further feedback on the reception of the program. Only a small proportion of the participants (18/232, 7.8%) reported that the SMS text messages were not useful, and 6.9% (16/232) reported that they did not receive the texts. This could be due to erroneous input of their mobile telephone number or because they blocked them (more details are discussed in the main paper of the study [19]). Incorrect mobile phone entries (either erroneously or fraudulently) or texts being blocked and marked as spam could be the reasons why participants did not receive the SMS text messages.

In terms of frequency, most participants (143/216, 66.2%) found the frequency of the mobile phone SMS text message program to be about right, with approximately one-third (70/216, 32.4%) reporting that they were too frequent, especially in the first 2 weeks, when they received 2 a day. This is a common issue in SMS text message interventions [28,29] and may differ depending on individual preferences. Similarly, some participants (14/216, 6.5%) reported blocking the messages because of their frequency. Previous research has highlighted that 20% of participants in practice trials requested to stop receiving SMS text messages [21]. Other reasons stated in the literature include not wanting or needing help or not having confidence in SMS text messages [30]. In phase 3, written feedback on the mobile phone SMS text message program revealed that almost half (21/47, 45%) of the recipients of the texts found them positive, for example, encouraging, supportive, and helpful; however, 34% (16/47) had a negative outlook on the program, and 15% (7/47) had a more mixed perspective. Our findings indicate that the codeveloped program was generally perceived as effective by the participants who provided feedback. However, a limitation of phase 3 is that only one-third (232/603, 38.5%) of those who received SMS text messages completed the follow-up survey. Thus, further research is needed to fully investigate the acceptability, usefulness, and utility of these SMS text messages and explore other combinations of messages or other formats (eg, app notifications) that may be effective. Finally, phase 3 revealed that those who rated the SMS text messages as useful were more likely to quit than those who did not. This could be because participants who found the mobile phone SMS text message program useful engaged more and recalled the information presented to them more often throughout the study period. Alternatively, it could mean that participants rated the messages as more useful because they were able to quit. Therefore, further investigation is needed regarding the association between usefulness ratings (1 of the constructs) and quit rates.

However, it is crucial to consider the rapidly changing e-cigarette landscape; the arrival of disposable vapes is changing the market and the traditional landscape of smoking cessation [31,32]. These devices have gained significant popularity as, unlike traditional vapes, they do not require recharging or refilling, hence minimizing the effort from the user. Although this mobile phone SMS text message program was developed with traditional vape devices in mind, it is important to consider the changes in the market and adapt the program according to needs.

Our work has limitations. Findings from phase 3 should be considered in the context of the data collection coinciding with the beginning of the COVID-19 pandemic, spanning the first and second lockdowns in England (March 2020 to October 2020). During that period, there were several changes—there were “quit for COVID” campaigns but also, in contrast, erroneous news reports suggesting that smoking protected one from the SARS-CoV-2 virus. Although our findings cannot be explained by the pandemic, there is a chance that the perceptions of people were affected by the news in the media regarding smoking, either in a positive or negative way. Finally, there were instances in which participants received SMS text messages when they did not have their vapes because of the delays posed by the COVID-19 pandemic. This would render the texts unhelpful as they were designed to coincide with the arrival of the e-cigarette device. Other limitations include automation errors during phase 3; there were cases in which participants did not receive the SMS text messages on time and cases in which participants did not receive the SMS text messages at all because of automation issues (eg, providing the wrong phone number). Further research is needed to examine the feasibility of the SMS text message program in helping smokers quit smoking by using vapes and test these SMS text messages in other forms such as notifications in mobile apps, which could lead to more interactive than passive engagement. Finally, the cost of running an SMS text message intervention should be considered by researchers as 2-way communication (participants responding to SMS text messages) will lead to more expensive interventions than 1-way communication (participants only receiving texts). The SMS text messages are available for other researchers to use for further evaluation and testing, either as a whole program or by selecting which theme or BCT they wish to address.

### Conclusions

This study underscores the potential benefits of coproduction in health research, including the ability to target community needs, engage community stakeholders, and promote person-centered practices. The codeveloped mobile phone SMS text message program has the potential to support smokers to quit smoking using a vape. However, the rapidly changing e-cigarette landscape suggests that further research is needed to investigate the effectiveness of the codeveloped SMS text message program by considering disposable vapes. Despite the aforementioned limitations, the program was perceived as effective by participants in phase 3, highlighting the potential benefits of incorporating community perspectives and expertise in the development of smoking cessation interventions.

## Appendix

Multimedia Appendix 1 Original list of 95 SMS text messages evaluated by smokers, ex-smokers, and vapers with the mean score across the 9 construct ratings (ordered from highest to lowest mean rating within each theme) and the Capability, Opportunity, and Motivation–Behavior construct.

Multimedia Appendix 2 Final set of 78 SMS text messages in the suggested order.

## Abbreviations

BCT: behavior change technique
COM-B: Capability, Opportunity, and Motivation–Behavior
