# Supplementary material for: Mobile Phone Text Messages to Support People to Stop Smoking by Switching to Vaping: Codevelopment, Coproduction, and Initial Testing Study
Source: JMIR Form Res. 2023 Sep 27;7:e49668. doi: 10.2196/49668 (PMC10568393; doi:10.2196/49668)
Supplement: Multimedia Appendix 1 [file formative_v7i1e49668_app1.docx]

**Multimedia Appendix 1.** Original list of 95 SMS text messages evaluated by smokers, ex-smokers, and vapers with the mean score across the 9 construct ratings (ordered from highest to lowest mean rating within each theme) and the Capability, Opportunity, and Motivation–Behavior (COM-B) construct.

| Primary theme and SMS text message example | | COM-B construct | People who rated the message, n | Values, mean^a^ (SD) |
| --- | --- | --- | --- | --- |
| **Smoking cessation support** | | | | |
|  | Vaping saves money | Capability—psychological | 38 | 5.77 (1.24) |
|  | You can save around £1,260 per year if you switch from smoking to vaping | Capability—psychological | 35 | 5.77 (1.32) |
|  | Need more support to stop smoking? Try visiting a Stop Smoking Service. Many now offer “vape friendly” services | Opportunity—physical and social | 41 | 5.37 (1.14) |
| **Social and practical support** | | | | |
|  | Quitting smoking is hard! You are doing really well. Don’t give up! | +Motivation—reflective | 23 | 6.14 (1.07) |
|  | Need more help and support? Talk to experienced vapers | Opportunity—social | 13 | 5.62 (1.63) |
|  | Need more advice and support? Try visiting a vape shop or an online forum for advice and support | Opportunity—physical and social | 16 | 5.41 (1.45) |
|  | If you can, seek out tips and advice from current vapers. There are lots of others who have switched to vaping and understand how it works | Opportunity—physical; motivation—reflective | 13 | 5.33 (1.37) |
|  | Want to talk to others who are quitting smoking? Try online forums where you will find lots of advice and support | Opportunity—social | 19 | 5.18 (1.29) |
|  | Reward yourself for quitting smoking. Treat yourself to a new vaporiser or something else | Motivation—reflective | 13 | 5.00 (1.12) |
| **Identity** | | | | |
|  | Don’t think you are too old and have smoked too many years to try vaping. Vaping can help adults of all ages quit smoking | Motivation—reflective | 19 | 6.15 (1.05) |
|  | Vaping is helping you to free yourself of smoking | Motivation—reflective | 18 | 6.12 (1.14) |
|  | It’s never too late to quit smoking! Even lifelong smokers have successfully switched to vaping | Motivation—reflective | 27 | 6.07 (1.22) |
|  | You have joined 1.7 million vapers in Great Britain who have moved away from smoking | Motivation—reflective | 20 | 5.92 (1.19) |
|  | Evidence suggests that vaping is the most popular form of quitting smoking | Motivation—reflective | 17 | 5.82 (1.17) |
|  | Try watching this short film for inspiration from vapers who made The Switch https[://](https://www)www.youtube.com/watch?v=GPxxBvf6hJU | N/A^b^ | 20 | 5.63 (1.36) |
|  | Most people prefer the smell of vape to tobacco smoke, so it is more acceptable to vape in public than to smoke | Opportunity—social | 28 | 5.47 (1.26) |
|  | Worried about gaining weight? If you vape and continue to use nicotine, you shouldn’t put on weight when you quit smoking | Capability—psychological | 24 | 5.36 (1.26) |
|  | Well done! You have joined 1.7m vapers in Great Britain who have switched to a lower risk nicotine product | Motivation—automatic | 24 | 5.34 (1.40) |
|  | Vaping doesn’t need to be a drama. Vapes come in different shapes, sizes and even cloud factor. Find the one(s) that fits with you Understandable | Capability—psychological | 24 | 5.25 (1.40) |
|  | Experiment by trying friends’ devices before you commit to buying your own | Opportunity—physical and social | 23 | 5.31 (1.13) |
|  | Love smoking? You can learn to love vaping instead! | Motivation—reflective | 16 | 5.16 (1.41) |
|  | Don’t listen to naysayers, this is your journey | Motivation—reflective | 30 | 4.84 (1.34) |
|  | Don’t want to be known as a “vaper”? Find a device that is small and discreet to avoid others making judgements | Motivation—reflective | 18 | 4.80 (1.34) |
|  | It is hard when you stop smoking as you might feel lonely or cut off from friends who continue to smoke. Try visiting a vape shop and making new friends who vape | Opportunity—social | 25 | 4.38 (1.47) |
|  | Some vapers like being part of a “vaping” group. You can find a tribe that suits you! | Motivation—reflective | 17 | 3.89 (1.74) |
| **Preventing lapse and relapse** | | | | |
|  | Don’t give up if you don’t like vaping straight away. There are plenty of other devices, nicotine strengths and flavours to try | Motivation—reflective; capability—psychological | 16 | 6.13 (0.97) |
|  | Every time you crave a cigarette go for your vape first and keep using it until the craving has passed | Motivation—automatic | 12 | 6.07 (1.28) |
|  | Whenever you have an urge to smoke, vape instead | Motivation—automatic | 21 | 6.05 (1.47) |
|  | The first flavour you try may not be your favourite—try another | Capability—physical | 17 | 5.95 (1.42) |
|  | Research suggests you are more likely to relapse to smoking if you have been drinking, so be prepared and don’t forget your vaporiser on nights out | Motivation—reflective | 22 | 5.90 (1.24) |
|  | Did you know almost 3 million people now use e-cigarettes in the UK? Over half of these have given up smoking. | Opportunity—social | 20 | 5.89 (1.03) |
|  | Craving a cigarette? Try vaping first | Motivation—reflective | 19 | 5.87 (1.26) |
|  | Don’t be afraid to use higher nicotine if you’re still craving a smoke. You can wean yourself off easier later | Capability—psychological | 15 | 5.81 (1.57) |
|  | If you’re craving a cigarette and vaping doesn’t fix it, try increasing your nicotine level | Capability—physical | 17 | 5.76 (1.18) |
|  | Social event coming up? Be prepared a higher strength e-liquid so you are not tempted to smoke if you have a craving | Motivation—reflective | 17 | 5.66 (1.34) |
|  | Try carrying a vape bag with extra coils, e-liquid and a charger with you. This could save you from smoking | Motivation—reflective; capability—psychological | 20 | 5.64 (1.06) |
|  | If you stop tasting one flavour of e-liquid, try switching to another, like mint | Opportunity—physical | 13 | 5.62 (1.26) |
|  | If you’re craving nicotine, don’t be worried, try using your device more often or go up a strength. | Capability—physical | 15 | 5.58 (1.62) |
|  | Don’t give up if you have a cigarette. Many people have gone on to quit successfully with vaping after a smoking lapse | Motivation—reflective | 17 | 5.52 (1.17) |
|  | If your partner smokes it is very hard to quit. Why not try switching to vaping together? | Motivation—reflective | 24 | 5.47 (1.11) |
|  | E-cigarettes need charging regularly. Try putting it on charge when you charge your mobile phone | Capability—physical | 17 | 5.46 (1.32) |
|  | E-cigarette not holding its charge? Contact your local vape shop or the research study investigator | Opportunity—physical | 24 | 5.43 (1.18) |
|  | Most cigarettes are associated with certain “triggers” (people, places, events). Try to recognise these and have your e-cigarette to hand instead | Motivation—automatic | 18 | 5.39 (0.85) |
|  | Still craving a cigarette? Just vape through it | Motivation—automatic | 14 | 5.13 (1.90) |
|  | Start with an all-in-one starter kit that won’t break the bank if you end up not liking it | Capability—physical | 17 | 5.26 (1.14) |
|  | Remember to charge your e-cigarette using the charger provided | Capability—physical | 12 | 5.26 (1.00) |
|  | Start with the highest nicotine level you can tolerate with the device you’re using | Capability—physical | 17 | 5.18 (1.28) |
|  | Drinking alcohol can be a weak link when trying to stop smoking. Try increasing your nicotine e-liquid content if you think alcohol could tempt you to smoke | Opportunity—physical; capability—psychological | 20 | 5.02 (1.15) |
|  | Struggling with cravings? More help and advice is available via the NHS stop smoking service | Opportunity—physical and social | 15 | 4.99 (1.42) |
|  | Getting bored of vaping? Try a new flavor! | Capability—psychological | 17 | 4.90 (1.32) |
| **Vaping vs smoking** | | | | |
|  | Don’t be worried if your e-cigarette never leaves your hand and you feel as if you’re constantly puffing. Vaping is not the same as smoking | Motivation—reflective | 19 | 6.38 (0.79) |
|  | Some people switch quickly, others take longer. Take as long as you need | Motivation—reflective | 34 | 6.04 (1.24) |
|  | Vaping is better for the environment. Secondhand vape produces less carcinogens than secondhand smoke | Capability—psychological | 31 | 5.95 (1.24) |
|  | You didn’t give up when you first started smoking so give vaping the same chance and be rewarded with better health | Motivation—reflective | 23 | 5.92 (1.26) |
|  | Don’t worry if you find yourself vaping lots. Vaping is different to smoking and you need to find patterns of vaping that best suit your needs | Motivation—reflective | 18 | 5.91 (1.21) |
|  | Vaping is better for the environment. Cigarette butts are messy and don’t biodegrade for hundreds of years. There is very little waste with vaping | Capability—psychological | 21 | 5.56 (1.03) |
|  | Don’t be concerned about vaping too much. Nicotine delivery is slower with e-cigarettes so you will need to vape more than you smoked | Capability—psychological; motivation—reflective | 23 | 5.55 (1.20) |
|  | Don’t be discouraged by subtle differences. Vaping isn’t exactly like smoking but in time you will adjust | Motivation—reflective | 26 | 5.50 (1.25) |
|  | Most people who switch from smoking to vaping are not successful the first time. Don’t give up trying | Motivation—reflective | 25 | 5.48 (1.31) |
|  | Don’t limit use of your e-cigarette, especially at first. It takes around 30 minutes of use to get the same nicotine as from one cigarette | Capability—psychological | 21 | 5.46 (1.37) |
|  | Vaping is different to smoking. Don’t worry if you seem to be vaping more | Motivation—reflective | 32 | 5.31 (1.09) |
|  | You may not get it right with your first vape but don’t give up. Just remember how awful it tasted and felt when you started smoking | Motivation—reflective | 33 | 5.30 (1.54) |
|  | Feeling stressed? Keep going with your vape. This feeling will pass and maybe try a different strength of nicotine | Motivation—reflective; capability—psychological | 18 | 4.86 (1.26) |
| **Practical vaping tips (equipment)** | | | | |
|  | Finding the right combination of device, liquid and flavours can take time. Don’t dismiss vaping straight away | Motivation—reflective | 18 | 6.38 (0.87) |
|  | Try before you buy: Many vape shops allow you to experiment with different products | Opportunity—physical | 22 | 6.20 (0.88) |
|  | Do not be embarrassed about using your vape in public | Motivation—automatic | 19 | 5.91 (1.41) |
|  | Don’t be afraid of trying lots of different flavours, and remember, your taste buds will change after you stop smoking and come back to life again | Motivation—reflective | 18 | 5.87 (1.47) |
|  | Dropped your vaporiser and smashed the tank? This can be replaced, so ask a vape shop for help | Opportunity—physical | 18 | 5.87 (1.00) |
|  | Some pubs and bars are vape friendly. Check with staff who might be happy to let you vape indoors | Opportunity—physical | 14 | 5.77 (1.25) |
|  | Try watching this short film for a practical intro to vaping for smokers: https[://](https://www)www.youtube.com/watch?v=fb1PfwEIoHY | N/A | 15 | 5.75 (1.03) |
|  | Experiencing a bad taste from your e-cigarette? Try changing the coil | Capability—psychological | 16 | 5.74 (1.14) |
|  | Try using a small waterproof bag to hold your vaping gear. Perfect for containing leaky tanks or e-liquid bottles | Capability—physical | 22 | 5.61 (1.14) |
|  | If your device isn’t working quite right, ask your local vape shop for technical support | Opportunity—physical | 16 | 5.60 (0.98) |
|  | Always use the approved charger for your vaporiser. It can be dangerous to use the wrong charger | Capability—psychological; opportunity—physical | 19 | 5.60 (0.67) |
|  | Not all e-cigarettes produce huge clouds. Try a different device or a different liquid if you want less vapour | Capability—psychological | 17 | 5.59 (1.24) |
|  | Concerned that you’re vaping all the time? Try increasing your nicotine level. Research shows people vape less with higher nicotine concentrations | Motivation—reflective; capability—psychological | 17 | 5.56 (1.38) |
|  | Device broken! Visit a vape shop and ask for help | Opportunity—physical | 21 | 5.41 (1.36) |
|  | Look for places that allow vaping. Many places are happy for people to vape, so don’t be shy to ask | Opportunity—social | 20 | 5.41 (1.07) |
|  | Coughing a lot? This is a common side effect of giving up smoking | Motivation—reflective | 29 | 5.38 (1.55) |
|  | If you don’t like using your device in public, try a smaller one for when you are out | Opportunity—physical | 23 | 5.30 (1.38) |
|  | Don’t keep your vaporiser in a pocket with loose change—this is a fire risk! | Capability—psychological | 22 | 5.22 (1.32) |
|  | Feeling more thirsty with vaping? This is perfectly normal and the extra fluids can be beneficial too | Motivation—reflective | 24 | 5.19 (1.56) |
|  | Remember to charge your e-cigarette using the charger provided | Capability—physical | 14 | 5.16 (1.03) |
|  | When refilling e-liquid, be careful not to overfill your device so you avoid leaks | Capability—physical | 20 | 5.16 (1.29) |
|  | If your e-cigarette “spits” liquid, just turn off the device and clean out excess liquid in the mouthpiece with some rolled up tissue | Capability—physical | 17 | 5.03 (1.18) |
|  | Try to keep your device away from water. You can wipe it clean with a cloth or tissue | Capability—physical | 20 | 5.02 (1.39) |
|  | More support to help you stay stopped from smoking here: https[://](https://www)www.uea.ac.uk/documents/246046/28183329/vaping+leaflet.pdf/aa23000b-d9df-2d9e-b617-9ab15464f61 | N/A | 16 | 4.45 (1.50) |
| **Health and safety** | | | | |
|  | Vaping is a safer, cleaner and much cheaper way of delivering nicotine | Capability—psychological | 17 | 6.37 (0.91) |
|  | Ignore people who say vaping is just as dangerous as smoking. Evidence suggests vaping is around 95% safer than smoking | Capability—psychological | 15 | 6.05 (1.26) |
|  | Remember to take your liquid with you in case you run out | Capability—physical | 17 | 5.96 (1.09) |
|  | There are far fewer cancer-causing particles in vape than in smoke. It is safer to vape around friends and family than to smoke | Capability—psychological | 17 | 5.70 (1.18) |
|  | People smoke for nicotine but it’s the harmful chemicals in smoke that damages health. Getting the nicotine without these chemicals will really help your health | Capability—psychological | 18 | 5.64 (1.51) |
|  | This short film by Public Health England shows how much safer e-cigarettes are than smoking: https[://](https://www)www.youtube.com/watch?v=RisBe5sLGPc | N/A | 19 | 5.61 (1.37) |
|  | Don’t panic if you read an article about the dangers of vaping. Research shows vaping is much less harmful than smoking | Capability—psychological | 16 | 5.32 (1.17) |
|  | Try watching this short film on top tips for vaping safety: https[://](https://www)www.youtube.com/watch?v=cuZRky79MMY | Ν/Α | 14 | 4.85 (1.73) |

^a^The constructs of positive or negative, enthusiastic or unenthusiastic, and interested or uninterested were reverse scored, so a higher score is more favorable.

^b^N/A: not applicable.
