# Supplementary material for: Mobile Phone Text Messages to Support People to Stop Smoking by Switching to Vaping: Codevelopment, Coproduction, and Initial Testing Study
Source: JMIR Form Res. 2023 Sep 27;7:e49668. doi: 10.2196/49668 (PMC10568393; doi:10.2196/49668)
Supplement: Multimedia Appendix 2 [file formative_v7i1e49668_app2.docx]

**Multimedia Appendix 2.** Final set of 78 SMS text messages in the suggested order.

| Number | Primary theme | SMS text message example | Target behavior | COM-B^a^ construct | BCT^b^ |
| --- | --- | --- | --- | --- | --- |
| 1 | Smoking cessation support | Your decision to buy an e-cig is brilliant, so feel proud of yourself. Remember, if there are tough times, you have what it takes—stay strong and stay confident.^c^ | e-Cigarette use | Motivation—reflective | Social support (unspecified; 3.1); verbal persuasion about capability (15.1) |
| 2 | Smoking cessation support | In 2019, 2 million ex-smokers were vaping in Great Britain. You can join them.^c^ | Smoking cessation | Motivation—reflective | Verbal persuasion about capability (15.1) |
| 3 | Practical vaping tips (equipment) | When you add liquid to your e-cig for the first time, it’s important to let it sit for a few minutes before taking a puff in order to avoid a nasty taste^c^ | e-Cigarette use | Capability—physical | Instructions on how to perform the behavior (4.1) |
| 4 | Practical vaping tips (equipment) | Coughing after taking an e-cig puff? Remember, inhaling vapour is different to inhaling smoke—you will learn how to do it.^c^ | Smoking cessation | Motivation—reflective; capability—psychological | Information about health consequences (5.1) |
| 5 | Practical vaping tips (equipment) | Remember to charge your e-cigarette using the charger provided | e-Cigarette use | Capability—physical | Instructions on how to perform the behavior (4.1) |
| 6 | Vaping vs smoking | Vaping is different to smoking. Don’t worry if you seem to be vaping more | e-Cigarette use/smoking cessation | Motivation—reflective | None identified^d^ |
| 7 | Preventing lapse and relapse | Every time you crave a cigarette go for your e-cig first and keep using it until the craving has passed | e-Cigarette use | Motivation—automatic | Behavior substitution |
| 8 | Identity | Don’t worry about how long you’ve been smoking to try vaping. Vaping can help adults of all ages quit smoking | e-Cigarette use | Motivation—reflective | Verbal persuasion about capability (15.1) |
| 9 | Practical vaping tips (equipment) | Do not be embarrassed about using your vape in public | e-Cigarette use | Motivation—automatic | Framing/reframing (13.2) |
| 10 | Smoking cessation support | Vaping saves money | e-Cigarette use | Capability—psychological | Information about social and environmental consequences (5.3) |
| 11 | Practical vaping tips (equipment) | Try watching this short film for a practical intro to vaping for smokers: https[://](https://www)www.youtube.com/watch?v=fb1PfwEIoHY | e-Cigarette use | Uncoded | Uncoded |
| 12 | Preventing lapse and relapse | Research suggests you are more likely to relapse to smoking if you have been drinking, so be prepared and don’t forget your vaporiser on nights out | e-Cigarette use/smoking cessation | Motivation—reflective | Problem-solving (1.2); information about antecedents (4.2) |
| 13 | Social and practical support | Quitting smoking is hard! You are doing really well. Don’t give up! | Smoking cessation | Motivation—reflective | Verbal persuasion about capability (15.1) |
| 14 | Vaping vs smoking | Feeling stressed? Keep going with your vape. This feeling will pass and maybe try a different strength of nicotine | e-Cigarette use | Motivation—reflective; capability—psychological | None identified |
| 15 | Smoking cessation support | You can save around £1,260 per year if you switch from smoking to vaping | e-Cigarette use/smoking cessation | Capability—psychological | Information about social and environmental consequences (5.3) |
| 16 | Practical vaping tips (equipment) | Try to keep your device away from water. You can wipe it clean with a cloth or tissue | e-Cigarette use | Capability—physical | None identified |
| 17 | Identity | Evidence suggests that vaping is the most popular form of quitting smoking | e-Cigarette use | Motivation—reflective | None identified |
| 18 | Practical vaping tips (equipment) | Don’t keep your vaporiser in a pocket with loose change—this is a fire risk! | e-Cigarette use | Capability—psychological | Information about social and environmental consequences (5.3) |
| 19 | Health and safety | Ignore people or organisations who say vaping is just as dangerous as smoking. Evidence suggests vaping is around 95% safer than smoking | Smoking cessation | Capability—psychological | Information about health consequences (5.1) |
| 20 | Preventing lapse and relapse | If you’re craving nicotine, don’t be worried, try using your device more often or go up a strength. | e-Cigarette use | Capability—physical | Instruction on how to perform the behavior (4.1) |
| 21 | Identity | Love smoking? You can learn to love vaping instead! | e-Cigarette use | Motivation—reflective | None identified |
| 22 | Practical vaping tips (equipment) | When refilling e-liquid, be careful not to overfill your device so you avoid leaks | e-Cigarette use | Capability—physical | Instructions on how to perform the behavior (4.1) |
| 23 | Vaping vs smoking | Don’t worry if you find yourself vaping lots. Vaping is different to smoking and you need to finds patterns of vaping that best suit your needs | e-Cigarette use | Motivation—reflective | Instructions on how to perform the behavior (11.1) |
| 24 | Practical vaping tips (equipment) | Experiencing a bad taste from your e-cigarette? Try changing the coil | e-Cigarette use | Capability—psychological | Instructions on how to perform the behavior (4.1) |
| 25 | Identity | It’s never too late to quit smoking! Even lifelong smokers have successfully switched to vaping | Smoking cessation | Motivation—reflective | Verbal persuasion about capability (15.1) |
| 26 | Practical vaping tips (equipment) | Coughing a lot? This is a common side effect of giving up smoking | Smoking cessation | Motivation—reflective | Information about health consequences (5.1) |
| 27 | Practical vaping tips | Make sure your tank is always at least half full otherwise you’ll get a horrible taste^c^ | e-Cigarette use | Capability—physical | Instructions on how to perform the behavior (4.1) |
| 28 | Preventing lapse and relapse | Craving a cigarette? Try vaping first | Smoking cessation | Motivation—reflective | Behavior substitution (8.2) |
| 29 | Preventing lapse and relapse | Try carrying a small bag with extra coils, e-liquid and a charger with you. This could save you from smoking | e-Cigarette use/smoking cessation | Motivation—reflective; capability—psychological | None identified |
| 30 | Preventing lapse and relapse | If you’re craving a cigarette and vaping doesn’t fix it, try increasing your nicotine level | e-Cigarette use/smoking cessation | Capability—physical | Problem-solving (1.2) |
| 31 | Practical vaping tips (equipment) | Try watching this short film on top tips for vaping safety: https[://](https://www)www.youtube.com/watch?v=cuZRky79MMY | e-Cigarette use | Uncoded | Uncoded |
| 32 | Health and safety | People smoke for nicotine but it’s the harmful chemicals in smoke that damages health. Getting the nicotine without these chemicals will really help your health | e-Cigarette use/smoking cessation | Capability—psychological | Information about health consequences (5.1) |
| 33 | Practical vaping tips | Remember to change your coil. If in doubt, put a new one in^c^ | e-Cigarette use | Capability—physical | Instructions on how to perform the behavior (4.1) |
| 34 | Vaping vs smoking | Don’t be discouraged by subtle differences. Vaping isn’t exactly like smoking but in time you will adjust | e-Cigarette use | Motivation—reflective | Verbal persuasion about capability (15.1) |
| 35 | Health and safety | Remember to take your liquid with you in case you run out | e-Cigarette use | Capability—physical | Instructions on how to perform the behavior (4.1) |
| 36 | Preventing lapse and relapse | Don’t give up if you don’t like vaping straight away. There are plenty of other devices, nicotine strengths and flavours to try | e-Cigarette use | Motivation—reflective; capability—psychological | Instructions on how to perform the behavior (4.1) |
| 37 | Preventing lapse and relapse | E-cigarettes need charging regularly. Try putting it on charge when you charge your mobile phone—though never overnight | e-Cigarette use | Capability—physical | Instructions on how to perform the behavior (4.1) |
| 38 | Identity | Don’t listen to naysayers, this is your journey | e-Cigarette use | Motivation—reflective | None identified |
| 39 | Practical vaping tips (equipment) | Finding the right combination of device, liquid and flavours can take time. Don’t dismiss vaping straight away | e-Cigarette use | Motivation—reflective | Instructions on how to perform the behavior (4.1) |
| 40 | Practical vaping tips (equipment) | Feeling more thirsty with vaping? This is perfectly normal and the extra fluids can be beneficial too | e-Cigarette use | Motivation—reflective | Information about health consequences (5.1) |
| 41 | Health and safety | This short film by Public Health England shows how much safer e-cigarettes are than smoking: https[://](https://www)www.youtube.com/watch?v=RisBe5sLGPc | e-Cigarette use | Uncoded | Uncoded |
| 42 | Vaping vs smoking | Don’t limit use of your e-cigarette, especially at first. It takes around 30 minutes of use to get the same nicotine as from one cigarette | e-Cigarette use | Capability—psychological | Instruction on how to perform the behavior (4.1) |
| 43 | Preventing lapse and relapse | Drinking alcohol can be a weak link when trying to stop smoking. Try increasing your nicotine e-liquid content if you think alcohol could tempt you to smoke | e-Cigarette use | Opportunity—physical; capability—psychological | Problem-solving (1.1) |
| 44 | Practical vaping tips (equipment) | If your e-cigarette “spits” liquid, just turn off the device and clean out excess liquid in the mouthpiece with some rolled up tissue | e-Cigarette use | Capability—physical | Instructions on how to perform the behavior (4.1) |
| 45 | Health and safety | There is only a fraction of cancer causing particles in vape than in smoke. It is safer to vape around friends and family than to smoke | e-Cigarette use/smoking cessation | Capability—psychological | Information about health consequences (5.1) |
| 46 | Preventing lapse and relapse | Don’t be afraid of trying lots of different flavours, and remember, your taste buds will change after you stop smoking and come back to life again | e-Cigarette use/smoking cessation | Motivation—reflective | Instruction on how to perform the behavior (4.1); information about health consequences (5.1) |
| 47 | Health and safety | Don’t panic if you read an article about the dangers of vaping. Research shows vaping is much less harmful than smoking | e-Cigarette use | Capability—psychological | Information about health consequences (5.1) |
| 48 | Vaping vs smoking | Don’t be worried if your e-cigarette never leaves your hand and you feel as if you’re constantly puffing. Vaping is not the same as smoking | e-Cigarette use | Motivation—reflective | Instructions on how to perform the behavior (4.1) |
| 49 | Practical vaping tips (equipment) | Always use the approved charger for your vaporiser. It can be dangerous to use the wrong charger | Purchasing e-cigarette equipment | Capability—psychological; opportunity—physical | Instructions on how to perform the behavior (4.1) |
| 50 | Preventing lapse and relapse | Getting low on e-liquid? Remember to buy some more^c^ | e-Cigarette use | Opportunity—physical | Instruction on how to perform the behavior (4.1) |
| 51 | Vaping vs smoking | Most people who switch from smoking to vaping are not successful the first time. Don’t give up trying | Smoking cessation | Motivation—reflective | Verbal persuasion about capability (15.1) |
| 52 | Preventing lapse and relapse | Don’t be afraid to use higher nicotine if you’re still craving a smoke. You can wean yourself off easier later | e-Cigarette use | Capability—psychological | Instruction on how to perform the behavior (4.1); verbal persuasion about capability (15.1) |
| 53 | Identity | Try watching this short film for inspiration from vapers who made The Switch https[://](https://www)www.youtube.com/watch?v=GPxxBvf6hJU | e-Cigarette use | Uncoded | Uncoded |
| 54 | Practical vaping tips (equipment) | If your device isn’t working quite right, ask your local vape shop for technical support | e-Cigarette use | Opportunity—physical | Social support (practical; 3.2) |
| 55 | Vaping vs smoking | You may not get it right with your first vape but don’t give up. Just remember how awful it tasted and felt when you started smoking | e-Cigarette use/smoking cessation | Motivation—reflective | Verbal persuasion about capabilities (15.1) |
| 56 | Preventing lapse and relapse | Whenever you have an urge to smoke, vape instead | e-Cigarette use/smoking cessation | Motivation—automatic | Behavior substitution (8.2) |
| 57 | Practical vaping tips (equipment) | Look for places that allow vaping. Many places are happy for people to vape, so don’t be shy to ask | e-Cigarette use | Opportunity—social | Information about social and environmental consequences (5.3) |
| 58 | Preventing lapse and relapse | Don’t give up if you have a cigarette. Many people have gone on to quit successfully with vaping after a smoking lapse | e-Cigarette use | Motivation—reflective | Social comparison (6.2) |
| 59 | Health and safety | Vaping is a safer, cleaner and much cheaper way of delivering nicotine | e-Cigarette use | Capability—psychological | Information about health consequences (5.1) |
| 60 | Vaping vs smoking | You didn’t give up when you first started smoking so give vaping the same chance and be rewarded with better health | e-Cigarette use/smoking cessation | Motivation—reflective | Verbal persuasion about capabilities (15.1) |
| 61 | Social and practical support | Want to talk to others who are quitting smoking? Try online forums where you will find lots of advice and support | Smoking cessation | Opportunity—social | Social support (unspecified; 3.1) |
| 62 | Preventing lapse and relapse | If you stop tasting one flavour of e-liquid, try switching to another, like mint | Purchasing e-cigarette equipment | Opportunity—physical | Instruction on how to perform the behavior (4.1) |
| 63 | Vaping vs smoking | Don’t be concerned about vaping too much. Nicotine delivery is slower with e-cigarettes so you will need to vape more than you smoked | e-Cigarette use | Capability—psychological; motivation—reflective | Instruction on how to perform the behavior (4.1) |
| 64 | Preventing lapse and relapse | Most cigarettes are associated with certain “triggers” (people, places, events). Try to recognise these and have your e-cigarette to hand instead | e-Cigarette use | Motivation—automatic | Problem-solving (1.2) |
| 65 | Smoking cessation support | Need more support to stop smoking? Try visiting a Stop Smoking Service. Many now offer “vape friendly” services | Smoking cessation | Opportunity—physical and social | Social support (unspecified; 3.1) |
| 66 | Identity | Most people prefer the smell of vape to tobacco smoke, so it is more acceptable to vape in public than to smoke | e-Cigarette use | Opportunity—social | Information about social and environmental consequences (5.3) |
| 67 | Health and safety | Remember to buy your e-liquid from a reputable source.^c^ | Purchasing e-cigarette equipment | Capability—physical | Instructions on how to perform the behavior (4.1) |
| 68 | Practical vaping tips (equipment) | Not all e-cigarettes produce huge clouds. Try a different device or a different liquid if you want less vapour | Purchasing e-cigarette equipment | Capability—psychological | Information on how to perform the behavior (4.1); pharmacological support (11.1) |
| 69 | Identity | Don’t want to be known as a “vaper”? Find a device that is small and discreet to avoid others making judgements | e-Cigarette use | Motivation—reflective | None identified |
| 70 | Practical vaping tips | The first flavour you try may not be your favourite—try another | e-Cigarette use | Capability—physical | None identified |
| 71 | Practical vaping tips | Dropped your vaporiser and smashed the tank? This can be replaced, so ask a vape shop for help | Purchasing e-cigarette equipment | Opportunity—physical | Information on how to perform behavior (4.1) |
| 72 | Social and practical support | Need more help and support? Talk to experienced vapers | e-Cigarette use | Opportunity—social | Social support (unspecified; 3.1) |
| 73 | Social and practical support | Reward yourself for quitting smoking. Treat yourself to a new vaporiser or something else | Smoking cessation | Motivation—reflective | Self-reward (10.9) |
| 74 | Identity | Worried about gaining weight? If you vape and continue to use nicotine, you shouldn’t put on weight when you quit smoking | e-Cigarette use/smoking cessation | Capability—psychological | Information about health consequences (5.1) |
| 75 | Vaping vs smoking | Vaping is better for the environment. Secondhand vape won’t harm people around you but remember, it’s not to everybody’s taste | e-Cigarette use | Capability—psychological | Information about social and environmental consequences (5.3); information about health consequences (5.1) |
| 76 | Preventing lapse and relapse | Getting bored of vaping? Try a new flavour! | e-Cigarette use | Capability—psychological | Instructions on how to perform the behavior (4.1) |
| 77 | Identity | Vaping doesn’t need to be a drama. Vapes come in different shapes, sizes and even cloud factor. Find the one(s) that fits with you | e-Cigarette use | Capability—psychological | None identified |
| 78 | Smoking cessation support | We hope you are still using your e-cig. Don’t give up if you smoke. Many people have still gone on to quit with vaping even if it takes a few attempts^c^ | e-Cigarette use | Motivation—reflective | Social comparison (6.2) |

^a^COM-B: Capability, Opportunity, and Motivation–Behavior.

^b^BCT: behavior change technique.

^c^These SMS text messages were newly added at stage 5.

^d^The pharmacological support BCT (11.1) applies to all texts in which e-cigarette use is the behavior and, therefore, has not been added for each one. EV, CN, and FN, who examined the SMS text messages, have completed a BCT workshop or web-based training.
